# Efficient genetic manipulation of the NOD-*Rag1-/-IL2RgammaC-null* mouse by combining *in vitro* fertilization and CRISPR/Cas9 technology

**DOI:** 10.1038/srep05290

**Published:** 2014-06-17

**Authors:** Feng Li, Dale O. Cowley, Debra Banner, Eric Holle, Liguo Zhang, Lishan Su

**Affiliations:** 1 Lineberger Comprehensive Cancer Center, Department of Microbiology and Immunology, University of North Carolina at Chapel Hill, Chapel Hill, NC 27599, USA; 2 Animal Models Core Facility, University of North Carolina at Chapel Hill, Chapel Hill, NC 27599, USA; 3 TransViragen, Inc., Research Triangle Park, NC 27709, USA; 4 Key Lab of Infection and Immunity, Institute of Biophysics, Chinese Academy of Sciences, Beijing 100101, China; 5 Department of translational medicine, Department of surgery, Department of medicine, the first hospital, Jilin University, Changchun 130061, China

## Abstract

Humanized mouse models have become increasingly important and widely used in modeling human diseases in biomedical research. Immunodeficient mice such as NOD-*Rag1*-/-*IL2RgammaC-null* (NRG) or NOD-*SCID*-*IL2RgammaC-null* (NSG) mice are critical for efficient engraftment of human cells or tissues. However, their genetic modification remains challenging due to a lack of embryonic stem cells and difficulty in the collection of timed embryos after superovulation. Here, we report the generation of gene knockout NRG mice by combining *in vitro* fertilization (IVF) and CRISPR/Cas9 technology. Sufficient numbers of fertilized embryos were produced through IVF, and a high rate of *Fah* gene targeting was achieved with microinjection of Cas9 mRNA, gRNA and single strand oligonucleotide DNA (ssDNA) into the embryos. The technology paves the way to construct NRG or NSG mutant mice to facilitate new humanized mouse models. The technology can also be readily adapted to introduce mutations in other species such as swine and non-human primates.

Small animal models are valuable tools to decipher the mechanism of gene function, disease and to test new therapeutics *in vivo*. However, translation of these studies to the clinic has met with limited success due to the species-specific differences between rodents and humans. Non-human primates serve as the best alternative to model human disease. Nevertheless, the prohibitively high cost and ethical concerns have limited their wide use. Recently, the ban on use of chimpanzees in biomedical research in Europe and the United States National Institutes of Health highlights the urgent need of *in vivo* models with the closest relevance to human, such as immunodeficient mice engrafted with human cells, tissues and organs. Immunodeficient mice with no T, B and NK cells are permissive as recipients for human cell engraftment and have been widely employed in investigation of human diseases including human-specific infections (as reviewed in^1^,^2^) such as virus infection (HIV, EBV, Dengue Virus, HBV, HCV), bacterial infection, protozoan infection; and in regenerative medicine, such as allograft (skin, liver, islet)^3^, organ regeneration^4^,^5^, and drug evaluation^6^.

Among the available models, immunodeficient mice with the NOD genetic background, such as NRG and NSG, have shown the highest permissiveness for human cell engraftment^7^. One major determinant of NOD mice is the unique cross-reactive activity of the NOD SIRP-α with its human receptor CD47, which reduces human rejection by NOD macrophages^8^. However, the limited availability of embryonic stem (ES) cells from immunodeficient and NOD mice^9^,^10^,^11^,^12^,^13^ and the difficulty of isolating embryos by natural mating^14^ have prohibited direct genetic manipulation to construct new NOD mutant models. As a result, all current knockout or transgenic NOD mice have been created by back crossing mutant mice from other genetic backgrounds, which is a time consuming (about 2 years) and labor intensive process. Although humanized mice with human blood and immune cells have been significantly improved in recent years, additional humanization in other organs such as liver remains challenging^15^,^16^.

Fumarylacetoacetate hydrolase (*Fah*) encodes the last enzyme in the tyrosine catabolism pathway, and Fah deficiency causes hepatocyte death through toxin accumulation^17^. Human hepatocytes can robustly repopulate the Fah mutant mouse liver in the FRG (*Fah*-/-*Rag2*-/-*IL2g*-/-) mouse^18^,^19^, which have been used to model human metabolism, liver injury, gene regulation, drug toxicity, and hepatotropic infections (reviewed in^20^). However, the role of the human immune system in these processes cannot be investigated because the current FRG mice in the C57/B6 background only poorly support the engraftment of human hematopoietic stem cells. More importantly, the mouse *Sirpa* gene is genetically linked with the *Rag1* and *Rag2* genes, making it extremely difficult to derive NRG-*Fah* mutant mice with the NOD *Sirpa* allele by genetic crossing. The ability to introduce additional mutations directly in embryos from NRG or NSG mice would greatly facilitate further improvements in humanized mouse models.

Novel approaches, such as zinc finger nuclease (ZFN), transcription activator-like effector nucleases (TALENs), and CRISPR/Cas9 nucleases, have greatly accelerated the production of genetically modified mice using direct injection of DNA or mRNA into one-cell stage embryos^21^,^22^,^23^,^24^,^25^, allowing construction of knockout mice by directly manipulating a small number of embryos.

Here, we report a strategy to directly inactivate the *Fah* gene in NRG mice by combining IVF and CRISPR/Cas technologies. With IVF, sufficient numbers of fertilized embryos were obtained for microinjecting gRNA, Cas9 mRNA and single strand DNA (ssDNA). A high success rate (>80%) of allele modifications was detected in the offspring, and the modified alleles were germline transmissible. Germline transmissible NRG-*Fah* mutant mice were generated in only 16 weeks. We have thus established a protocol to rapidly introduce genetic mutations directly in NRG mice, which can also be adapted to other mouse strains and other species.

## Results

### Generation of gRNAs targeting the mouse *Fah* gene

**Figure 1:**
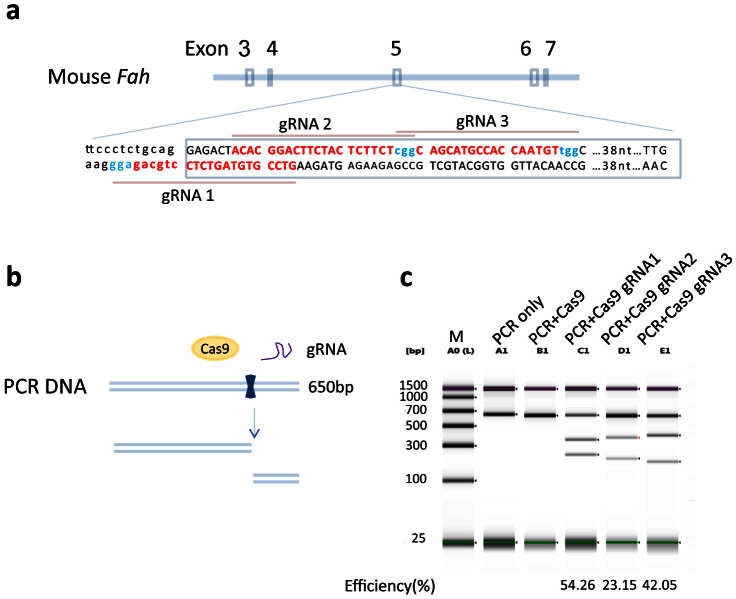
gRNAs targeting *Fah* exon5 can efficiently direct Cas9 cleavage of target DNA. (a) gRNA sequences against *Fah* exon5. The exon 3, 4, 5 and 6 region of the mouse *Fah* gene is shown. The exon5 sequence (upper case) and part of the upstream intron sequence (lower case) are shown with 3 gRNA sequences (labeled in red), and the PAM sequence NGGs in turquoise. (b) and (c) gRNA cleavage efficiency *in vitro*. (b) Schematic diagram of gRNA mediated Cas9 DNA cleavage. The PCR DNA product containing the gRNA target sites, Cas9 nuclease protein and gRNA are mixed. The Cas9/gRNA complex cuts the PCR product into two fragments. (c) The cleaved DNA was resolved on TapeStation. A0(L), DNA ladder; A1, PCR DNA alone; B1, PCR DNA + Cas9 nuclease; C1, D1 and E1, PCR DNA + Cas9 nuclease + gRNA1 (C1), gRNA2 (D1) and gRNA 3 (E1). Full-length gel is shown. The gRNA mediated cutting efficiency is shown below each lane.

The murine *Fah* gene has 14 exons and encodes a 419 amino acid hydrolase responsible for the degradation of fumarylacetoacetic acid to fumarate and acetoacetate in the last step of the tyrosine catabolism pathway. To introduce a null mutation in the *Fah* gene in the NRG background, exon 5 was chosen as previously reported^26^. Three gRNAs targeting the exon 5 splice donor sequence and coding region were selected (Fig. 1a). Two complementary oligo DNAs were annealed and inserted into BsaI linearized vectors pT7-BsaI-gRNA-Kan. The gRNAs were generated *in vitro* with T7 RNA polymerase using the linearized DNA as template. The function of each guide RNA was tested *in vitro* by mixing the gRNA, Cas9 protein and a PCR segment (650 bp) covering the three gRNA target sites. As illustrated in Fig. 1b, all three gRNAs can direct Cas9 cleavage of their target sites *in vitro*. The cutting efficiency was analyzed using a DNA TapeStation (Fig. 1c). No cleavage was observed when only Cas9 was added. However, DNA cleavage was observed when each gRNA was added, indicating all three gRNAs were functional. The cutting efficiencies of gRNA1, 2 and 3 are 54.26%, 23.15% and 42.05%, respectively.

### Production of NRG embryos using *in vitro* fertilization and genetic manipulation by pronuclear microinjection

**Figure 2:**
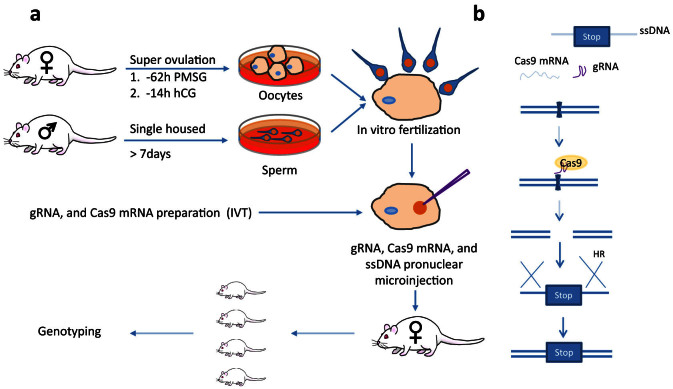
Strategy to generate NRG *Fah* knockout mice. (a) Schematic illustration of IVF and pronuclear microinjection. Female NRG mice are superovulated with PMSG and hCG followed by oocyte collection. Sperm is collected from male NRG mice. The oocytes and sperm are incubated to generate fertilized eggs and embryos, which are then microinjected with gRNA, Cas9 mRNA and ssDNA in the pronucleus. The injected embryos are then transferred into pseudopregnant surrogate mothers. Mouse pups are genotyped. (b) Mechanism of gRNA, Cas9 mRNA, and ssDNA mediated *Fah* gene knockout. Cas9 mRNA is translated into Cas9 protein after microinjection. The Cas9/gRNA complex binds the genomic DNA and generates DSBs. The ssDNA contains homologous sequence spanning the double strand break sites with ~50 bp on each side, two stop codons and a BamHI site. The ssDNA can be used as a template for homologous recombination to introduce the stop codons and BamHI site. The mouse is drawn by the authors (F.L. and L.S.), using Adobe Photoshop and Adobe Illustrator.

It is challenging to get fertilized eggs or embryos from NRG mice through natural mating. Experienced NRG stud males failed to plug NRG females that had been superovulated using a standard PMSG/HCG hormone regimen (data not shown). The failure to copulate was confirmed by the observation that only unfertilized oocytes were harvested from the females, although oocyte numbers were indicative of successful superovulation. Failure of obtaining fertilized embryos by natural mating was also reported in the NOD-SCID mouse^14^. To overcome the lack of plugging and fertilization by NRG males, we used IVF to produce embryos from NRG mice (Fig. 2a). IVF is a widely used assisted reproductive technology, and has been shown to be highly effective to produce healthy mice^27^. Male NRG mice were individually caged for 10 days before sperm isolation. Female NRG mice were dosed with 5 IU PMSG, followed 48 hours later by 5 IU of HCG. Oocytes were isolated 14 h after HCG injection, were mixed with capacitated sperm and incubated *in vitro* for 6 h. Oocytes isolated from females from 4–10 weeks old could be efficiently fertilized *in vitro* (Table 1). However, fewer oocytes were obtained from 4 week old mice than 6–10 wk old animals. After identifying the fertilized embryos, the gRNA, Cas9 mRNA and ssDNA were delivered to the pronucleus of fertilized embryos still in one cell stage by microinjection using a continuous flow injection mode. Surviving embryos were subsequently implanted into the oviducts of pseudo-pregnant Swiss Webster recipient females. The resulting offspring were screened for mutated *Fah* alleles.

Fig. 2b illustrates the *Fah* gene editing procedures. Cas9 nuclease is translated from injected mRNA using the cell translation machinery. The Cas9/gRNA complex finds and cleaves the gRNA target site, creating a DNA double strand break (DSB). DSBs can be repaired by cellular repair mechanisms including error-prone non-homologous end joining (not shown) and homologous recombination (HR). The introduced ssDNA with homology to the sequences flanking the Cas9/gRNA target site can be used as a template for HR-mediated repair, and has been demonstrated to be a better alternative to the traditional longer targeting vectors in mediating the homologous recombination^22^,^28^. The ssDNA contains two successive in-frame stop codons and a BamHI site (not shown). After homologous recombination the stop codons and BamHI site will be incorporated into the genomic DNA, generating a truncated FAH as reported^26^.

### Identification of NRG mice with the genetically edited *Fah* alleles

**Figure 3:**
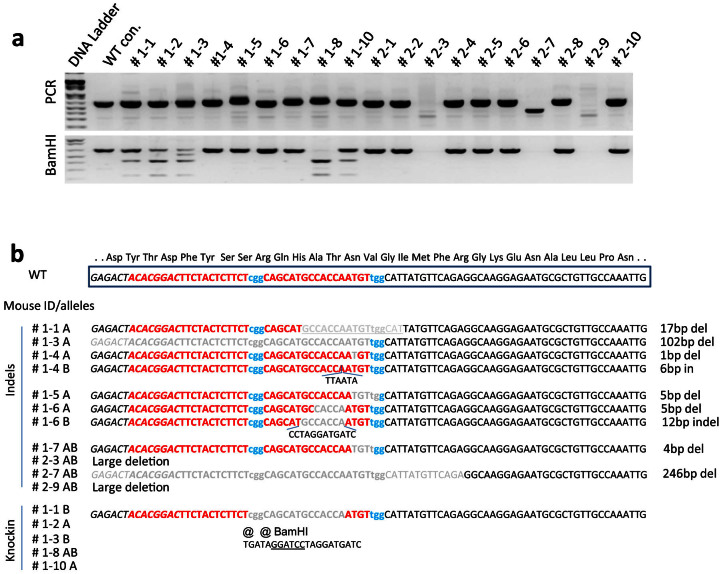
Genotyping and sequence analysis of founder mice. (a) PCR results of founder mice. Mice from two microinjection sessions were genotyped by PCR amplification and BamHI digestion. PCR and BamHI digestion products were electrophoresed in 1.2% argorose gel. Mice from the first injection session (wild type Cas9 mRNA) were labeled #1-1 to #1-10, and mice from the second injection session (mutant D10A Cas9 mRNA) were labeled #2-1 to #2-10. Wild type mouse genomic DNA was used as negative control (WT con). Cropped gels are shown. Full-length gels are provided for review in the supplementary file. (b) Sequence analysis of the mutated *Fah* alleles. The wild type sequence of exon5 is shown on top, with the encoded amino acids indicated above the sequence. The gRNA targeting sites are shown in red letters and PAM NGGs in turquoise. The mutant alleles of each mouse are labeled with A and B, following the mouse ID number. The indels for each mutated allele from different mice are shown in the middle. The deleted sequences are marked in gray, and the inserted sequences are shown below the wild type sequence. The deletion or insertion length of each indel is shown on the right. The *Fah* mutant alleles with the knock-in sequence are shown in the lower panel. Only one sequence is shown for the stop codon (@) and BamHI insertion. Indel, insertion and deletion; in, insertion; del, deletion.

We tested *Fah* mutagenesis using both Cas9 wild type (WT) nuclease and the Cas9 nickase mutant (D10A)^29^ (Table 2). The Cas9 (WT) nuclease can utilize a single gRNA to mediate site-specific DSBs, which can be repaired through either non-homologous end joining (NHEJ) or homology-directed repair (HDR). The Cas9 (D10A) mutant is a nickase version of Cas9 that can only cause a DSB when provided with two gRNAs targeting sites near each other on opposite strands. These DSBs can be repaired through NHEJ or HDR, while any off-target cleavage activity by Cas9 (D10A) results in single strand nicks that are typically repaired through high-fidelity HDR, resulting in lower risk of germline transmissible off-target mutagenesis^29^,^30^. In the first injection session, gRNA3 and Cas9 (WT) mRNA were injected, and 201 injected embryos were transferred into 8 pseudopregnant surrogate females. 4 females gave birth to a total of 11 pups (including 2 dead pups). In the second injection, gRNA1, gRNA3 and Cas9 (D10A) mRNA were injected, and 147 embryos were transferred into 6 pseudopregnant recipient females. 2 females gave birth to 11 pups (including 1 pup that died and was not recovered for genotyping). Genomic DNA was extracted for PCR amplification and the purified PCR products were then subjected to BamHI digestion (Fig. 3a). Of the 9 pups (#1-1 to #1-10) from the first microinjection, 5 had insertion of the BamHI site, including homozygous insertion in mouse #1-8. From the second injection, none of the 10 pups had BamHI site insertion. However, 3 mice from injection 2 showed evidence of large biallelic deletions at the *Fah* locus. Mouse #2-7 gave an amplicon approximately 250 bp shorter than expected, and no amplicon was obtained from mouse #2-3 and #2-9, even when the PCR amplicon was extended to cover 400 bp each side from the cutting sites (data not shown). Together, these data indicate that both Cas9 (WT) and Cas9 (D10A) gave efficient editing at the *Fah* locus in NRG embryos.

To define the mutations in the *Fah* gene, the PCR products were subjected to DNA sequencing. The two alleles of each mouse were labeled with A and B (Fig. 3b). One allele of mouse #1-1 had the knock-in stop codons and BamHI site, while the other allele had a 17 bp deletion. One allele of mouse #1-2 and #1-10 had the knock-in stop codons and BamHI site, while the other was wild type. Mouse #1-3 had 3 alleles detectable by sequencing: one was the knock-in stop codons and BamHI site, another was a 102 bp deletion, and the third was wild type. The BamHI digestion for this animal also showed an additional band under the wild type product, which was presumably the band with the 102 bp deletion (Fig. 3a). The PCR digestion and sequence results confirmed that mouse #1-3 was a mosaic mouse. Both alleles of mouse #1-8 had the knock-in sequence. Although mice #1-4, #1-5, #1-6, and #1-7 didn't have the knock-in sequence, they had indels (insertion and/or deletion mutations), indicating their genomic DNA had been cut by Cas9. One allele of mouse #1-4 had a 1 bp deletion, and the other allele had a 5 bp insertion. One allele of mouse #1-5 had a 5 bp deletion, and the other allele was wild type. One allele of mouse #1-6 had a 5 bp deletion, and the other allele was a replacement of 7 bp with a 12 bp insertion. Both alleles of mouse #1-7 had a 4 bp deletion. Sequence results confirmed that mouse #2-7 had a 246 bp deletion spanning exon5 and its upstream intron. The alleles of mice #2-1, #2-2, #2-4, #2-5, #2-6, #2-9, #2-10 from the second injection were wild type without any modification.

### Successful germline transmission of the knockout *Fah* allele

**Figure 4:**
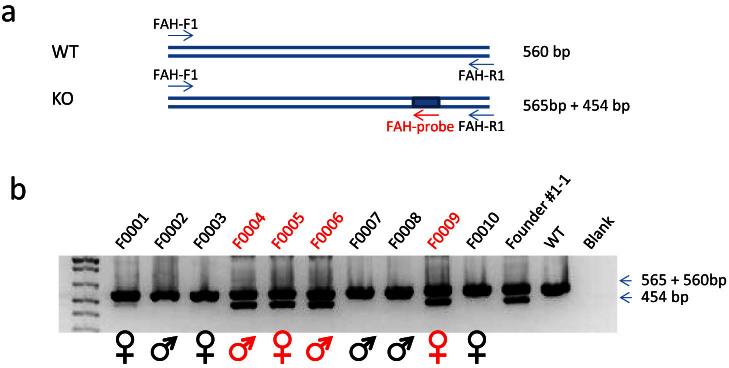
Germline transmission of the *Fah* mutant allele. (a) Primers designed to genotype the *Fah* mutant allele by PCR. Primers FAH-F1, FAH-R1 and FAH-probe are used in the same PCR reaction. Primers FAH-F1 and FAH-R1 amplify a 560 bp segment from the WT and 565 bp from the STOP-BamHI knock-in allele. The primers FAH-F1 and FAH-probe, specifically targeting the knock-in sequence, only amplify a 454 bp segment. (b) PCR genotyping results of F1 generation mice. The mouse numbers, from F0001 to F0010, are shown above each lane. Founder #1-1 and wild type mouse genomic DNA serve as positive and negative controls, respectively. Blank is a reaction without any DNA template. The amplicon size is shown on the right. The gender of each mouse is shown below each lane. ♀, female; ♂, male. Left lane, DNA ladder. Cropped gel is shown. Full-length gel is provided for review in the supplementary file.

Founder mouse #1-1 was chosen to breed with male NRG mice to test whether the edited *Fah* allele is germline transmittable. A specific primer (FAH-Probe) targeting the edited sequence introduced by ssDNA was used to amplify the knockout allele. As shown in Fig. 4a, the primers FAH-F1 and FAH-R1 can amplify 560 or 565 bp segments using the wild type and knockout alleles as templates, respectively, but the FAH-F1 and FAH-Probe can only amplify the knockout allele to generate a short amplicon (454 bp). Mouse #1-1 had normal litter size with typical Mendelian inheritance (Fig. 4b). Of the 10 pups, 2 females and 2 males had the *Fah* knockout allele, and 3 females and 3 males had only the wild type allele. This result demonstrates that the *Fah* knockout alleles generated in NRG mice are germline transmissible.

## Discussion

Both NRG and NSG mice are widely used for engrafting human normal and tumor cells (reviewed in ref. 1,31), since they can support efficient engraftment of human hematopoietic stem cells and human immune system maturation^7^,^8^. However, significant barriers have impeded the introduction of additional genetic modifications in NRG and NSG mice. The difficulty of deriving germline-competent ES cell lines from NOD strains, including NRG and NSG, has hindered efforts to perform gene targeting in these strains. Although more robust protocols have recently been developed for ES cell derivation from NOD or other “non-permissive” strains^11^,^12^,^13^, such ES cells are not widely available and their utility for gene targeting and germline transmission remain to be fully established. Transgenic manipulation of NRG and NSG mice through embryo microinjection is also difficult due to the poor embryo yields produced through natural mating^14^,^32^. Thus, nearly all current NOD transgenic and NOD knockout mice were derived by backcrossing the mutant genes or transgenes from other mouse strains. To our knowledge, our work represents the first report of the successful generation of genetically modified mutant mice in NOD-derived immunodeficient mice using RNA-guided Cas9 nucleases and IVF. The manipulation of mammalian genomes has been significantly facilitated by the recent development of novel approaches to target DNA breaks to specific loci using nucleases such as ZFNs, TALENs and CRISPR^21^,^22^,^23^,^24^,^25^. The easy design and high targeting efficiency of CRISPR/Cas9 technology enables the fast generation of knock-in and knockout mice by directly manipulating a small number of embryos using microinjection^25^. To overcome previous limitations of the NRG strain, IVF was conducted and combined with the CRISPR/Cas9 gene editing technology. The relatively low number of embryos required for successful gene modification by microinjection was easily obtained using our IVF approach (Table 2). The CRISPR/Cas9 technology gave efficient genetic modification in NRG embryos, enabling production of a novel mutant strain in the NRG background in a few weeks. This approach has several advantages compared to ES cell approaches, including shorter timelines, reduced costs and higher probability of germline transmission.

*Fah* knockout mice in other mouse backgrounds can support robust human hepatocyte repopulation in the chimeric liver, which have been successfully used to model human metabolism, liver injury, gene regulation, drug toxicity, and human hepatotropic infections^18^,^19^,^20^. Crossing the *Fah* mutation to NRG or NSG background would require at least 2 years (10 backcrosses, and 1 intercross, 10 week/cross cycle), which is a time consuming and labor intensive procedure. More importantly, since the NOD Sirp-α gene is genetically linked with *Rag*1/2 on chromosome 2, generation of NOD-*Fah*-/-*Rag2*-/-*IL2rg*-/- mice by backcrossing *Fah*-/-*Rag2*-/-*IL2rg*-/- mice with NOD mice would require a rare meiotic crossover event to combine the NOD Sirpα and Rag mutant alleles. The animals obtained after 10 backcross generations would therefore be unlikely to have the NOD *Sirpα* allele^33^, resulting in continued poor human cell repopulation activity. Our system avoids this issue by generating the *Fah* mutant allele directly in the NSG background.

In our experiments, Cas9 (WT) was much more efficient than Cas9 (D10A) at inducing mutations in the *Fah* gene. The Cas9 (WT) protein creates a double strand break (DSB) at the gRNA target site, but may have a risk of off-target mutagenesis. The Cas9 D10A mutant, on the other hand, is converted to a nickase, and only makes nicks on one strand of the target DNA, limiting DSBs to sites with adjacent gRNA targets on opposite strands. While the nickase strategy is a promising way to reduce the risk of off-target activity, it was much less efficient than Cas9 (WT) in our experiments (100% mutation efficiency with Cas9 (WT) vs 30% with Cas9 (D10A)). Moreover, we did not obtain any mice with homologous insertion of the oligonucleotide donor using the Cas9 nickase approach, while over 50% of mice obtained from the Cas9 (WT) injections had the knock-in event. This suggests that the nickase approach will require additional development to define conditions for efficient induction of homologous recombination in mouse embryos.

In summary, we have established a technology to derive embryos by IVF combined with a highly effective CRISPR/Cas9-based system to produce targeted mutations in immunodeficient NRG mice. Importantly, the same procedures can be readily applied to edit the genomes of other mammals such as swine and non-human primates.

## Methods

### Ethics statement

All animal experiments were conducted following NIH guidelines for housing and care of laboratory animals and in accordance with the University of North Carolina at Chapel Hill in accordance with protocols approved by the institution's Institutional Animal Care and Use Committee (protocol numbers 13-088 and 12-250).

### Plasmid construction

The gRNAs were identified using the online software (http://www.genome-engineering.org/crispr/?page_id=41). Complementary oligos for each target sequence were heated at 95°C for 5 mins, and annealed by decreasing 0.20°C/second to 16°C using a PCR machine (BioRad). Then, the short double strand DNA fragment were ligated into BsaI linearized pT7-BsaI-gRNA-Kan. Primers for each target RNA are as follows: gRNA1 (5′- TATAGTCCGTGTAGTCTCCTGCAG-3′, 5′-AAACCTGCAGGAGACTACACGGAC-3′), gRNA2(5′- TATAGCACGGACTTCTACTCTTCT-3′, 5′-AAACAGAAGAGTAGAAGTCCGTGC-3′), and gRNA3 (5′- TATAGGGCAGCATGCCACCAATGT-3′, 5′-AAACACATTGGTGGCATGCTGCCC-3′). Plasmids were prepared using MiniPrep kit (Qiagen) and MidiPrep kit (Qiagen). The sequence was confirmed by sequencing (Eton Bioscience Inc.).

### mRNA and gRNA preparation

gRNAs were generated through *in vitro* transcription reaction using T7 High Yield RNA Synthesis Kit (NEB #E2040S). Briefly, 1 μg DraI linearized template DNA was used in a 20 μl reaction following kit guidelines for short RNA transcripts. The reaction was incubated at 37°C overnight, followed by DNase I (RNase-free) digestion for 15 mins at 37°C. The gRNAs were then purified using an RNEasy Column following guidelines for short RNA purification (Qiagen).

Capped and polyadenylated Cas9 and Cas9-D10A mRNAs were prepared through *in vitro* transcription reaction using mMESSAGE mMACHINE T7 ULTRA KIT (Life Technologies AM1345). Briefly, to generate Capped mRNA. 1 μg hCas9-T7 or hCas9-D10A-T7 linearized plasmid DNA was added into a 20 μl reaction containing 1× NTP/ARCA, 1× T7Reaction Buffer, and 2 μl T7 Enzyme. The reaction was incubated at 37°C for 1 h, followed by addition of 1 μl TURBO DNase and digestion at 37°C for 15 mins. To add polyA tails to the Capped mRNA, the 20 μl reaction mix from step1 was mixed with Nuclease-free Water (36 μl), 5× E-PAP Buffer (20 μl), 25 mM MnCl2 (10 μl), ATP Solution (10 μl), and E-PAP (4 μl) to a final reaction volume of 100 μl. The reaction was incubated at 37°C for 30–45 mins. The capped & polyadenylated RNA was purified by LiCl precipitation and resuspended in microinjection buffer (5 mM Tris, 0.1 mM EDTA, pH 7.5).

### Mouse genomic DNA extraction and PCR

The mouse gDNA was extracted from mouse toe or ear samples using the KAPA Mouse Genotyping Kit. Briefly, tissues around 2 mm^3^ were digestion in a 100 μl volume containing 88 μl PCR-grade H_2_O, 2 μl 10× KAPA express Extract buffer and 2 μl KAPA Express Extract Enzyme, at 75°C for 15 mins, followed by 95°C for 5 mins to inactivate the enzyme. Primers used for the PCR are as follows: FAH-F1, (5′-AATGGGAGAAATGAGGCTAAGGAGC-3′, FAH-R1 (5′-AGGGTCTTTGCTGCTGGGAAAAA-3′), FAH-R2(5′-TCATGCTGAGGGAACCAAAAGCC-3′), FAH-Probe(5′-TGAACATAATGCCAACATGATCATCC-3′).

### *In vitro* guide RNA test

His-tagged Cas9 protein was expressed in bacteria and purified using Ni-NTA agarose and gel exclusion chromatography (Protein Expression and Purification Core Facility, UNC Chapel Hill). 1 μg of a PCR segment (650 bp) covering the gRNA target site, 600 ng of gRNA obtained by *in vitro* transcription and 0.6 μg CAS9 protein were mixed in a 20 μl reaction with 1× NEB buffer 3 and 5× BSA (NEB). The reaction was incubated at 37°C for 1 h, followed by heat inactivation of Cas9 by adding loading dye and incubating at 80°C 10 mins. The cut DNA was electrophoresed in a TapeStation (Agilent), and the cutting efficiency was calculated by the following formula, cutting efficiency (%) = sum (cut large band + cut small band)/sum (uncut PCR DNA + cut large band + cut small band) × 100.

### Superovulation and *In Vitro* fertilization (IVF)

Female NRG mice (4–10 weeks of age) were superovulated by intraperitoneal injection with 5 IU pregnant mare serum gonadotropin (National Hormone & Peptide Program, NIDDK), followed 48 hours later by injection of 5 IU human chorionic gonadotropin (hCG, National Hormone & Peptide Program, NIDDK). The animals were sacrificed 14 hours following hCG administration and oviducts collected. Oocyte-cumulus complexes were released from the oviducts and placed in pre-equilibrated fertilization drops consisting of Cook's Media (Cook Medical Research).

Fresh sperm was isolated from the epididymis and vas deferens of male NRG mice (5 months of age) into Cook's medium under mineral oil. Sperm capacitation was performed by incubation (37°C, 5% O_2_, 5% CO_2_, 90% N_2_) in Cook's medium for 1 hour.

Fertilization was carried out in Cook's medium under mineral oil by transferring oocyte-cumulus complexes to fertilization drops containing the activated sperm and incubating for 6 hours (37°C, 5% O_2_, 5% CO_2_, 90% N_2_). After incubation, the oocytes were washed in fresh Cook's medium to remove excess sperm, transferred to Cook's medium and incubated until microinjection performed.

### Pronuclear microinjection and transfer to pseudopregnant recipients

Fertilized embryos with visible pronuclei following IVF were selected for pronuclear microinjection and transferred to microinjection dishes containing M2 medium under mineral oil. The CRISPR/Cas reagent mixture was prepared by dilution of the components into injection buffer (5 mM Tris, 0.1 mM EDTA, pH 7.5) to obtain the following concentrations: 100 ng/μl Cas9 mRNA (wild-type or D10A), 50 ng/μl FAH guide RNA, 100 ng/μl single stranded donor oligonucleotide (5′- GGGTGTTCCCTCTGCAGGAGACTACACGGACTTCTACTCTTCTTGATAGGATCCTAGGATGATCATGTTGGCATTATGTTCAGAGGCAAGGAGAATGCGCTGTTGCCA-3′). The reagent mixture was introduced into the pronuclei of fertilized embryos by microinjection using a continuous flow injection mode. Surviving embryos were surgically implanted into the oviducts of pseudopregnant Swiss Webster recipient females. The resulting offspring were analyzed for editing of the *Fah* gene.

## Supplementary Material

Supplementary Information Supp information

## Figures and Tables

**Table 1:** Summary of *in vitro* fertilization (IVF)

Mouse age (weeks)	4	6	8	10
Female Number	3	9	6	7
Total oocytes	62	487	394	507
Fertilized embryos	45	173	160	156
Fertilization rate (%)	72.6	35.5	40.6	30.8
Embryos/mouse	15	19.2	26.7	22.3

**Table 2:** FAH knockout mice

					Mutant Alleles per Mouse/Total Mice Tested					
Gene	gRNA	Cas9	Transferred Embryos (Recipients/pregnancies)	Newborns (dead)	2 indel	1 indel	2KI	1KI	1indel +1 KI	0
FAH	gRNA3	WT Nuclease	211** (8/4)	11 (2)	3	1	1	2	2	0
	gRNA1 + gRNA3	D10A Mut Nickase	147 (6/2)	11 (1)	3	0	0	0	0	7

**201 injected embryos were transferred. One recipient received 11 injected and 10 non-injected embryos. This recipient did not produce any births.
