# Supplementary material for: Efficient genetic manipulation of the NOD-Rag1-/-IL2RgammaC-null mouse by combining in vitro fertilization and CRISPR/Cas9 technology
Source: Sci Rep. 2014 Jun 17;4:5290. doi: 10.1038/srep05290 (PMC4894429; doi:10.1038/srep05290)

Supplementary information

Efficient genetic manipulation of the NOD-*Rag1-/-IL2RgammaC-null* mouse by combining *in vitro* fertilization and CRISPR/Cas9 technology

Authors: Feng Li[1], Dale O. Cowley[2,3], Debra Banner[3], Eric Holle[2], Liguo Zhang[4],and Lishan Su[1,4,5*]

[1]Lineberger Comprehensive Cancer Center, Department of Microbiology and Immunology, University of North Carolina at Chapel Hill, Chapel Hill, NC 27599, USA

[2]Animal Models Core Facility, University of North Carolina at Chapel Hill, Chapel Hill, NC 27599, USA

[3]TransViragen, Inc., Research Triangle Park, NC 27709, USA

[4]Center for Infection and Immunity, Institute of Biophysics, Chinese Academy of Sciences, Beijing 100101, China

[5]Department of translational medicine, Department of surgery, Department of medicine, the first hospital, Jilin University, Changchun 130061, China

***Correspondence:** Correspondence should be addressed to L.S. (lsu@med.unc.edu), telephone: 919-966-6654; fax: 919-966-8212

Supp. Fig 3a. Supplementary full length DNA gel.

a

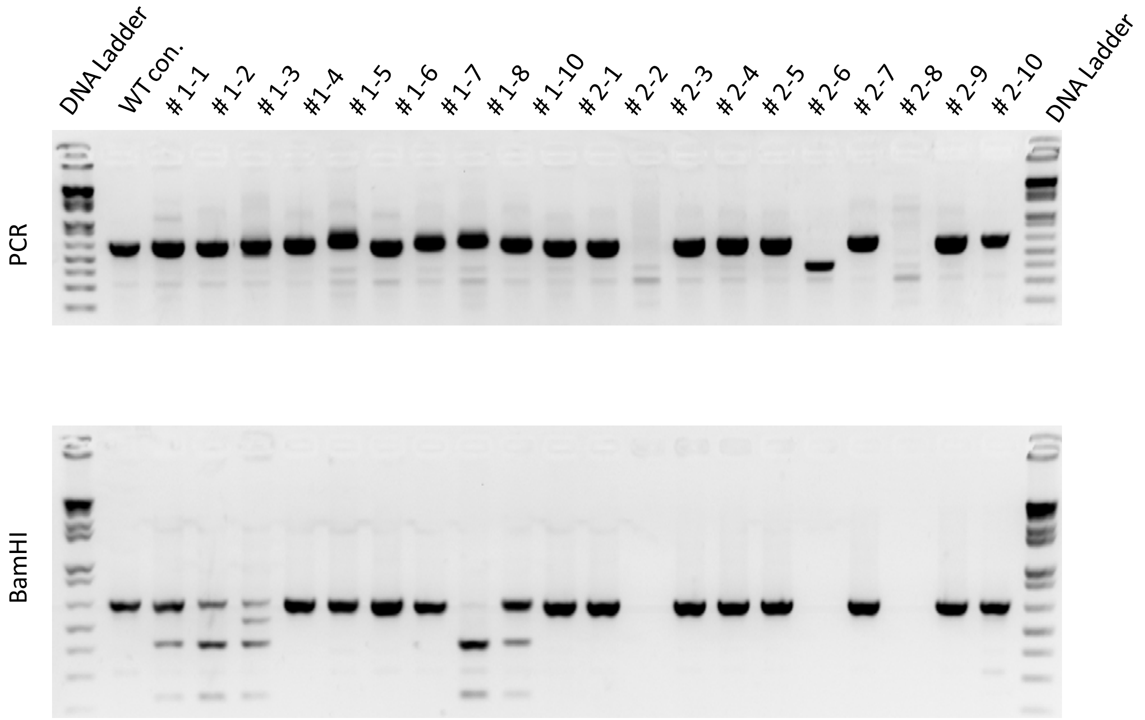

Supp. Fig 4b. Supplementary full length DNA gel.

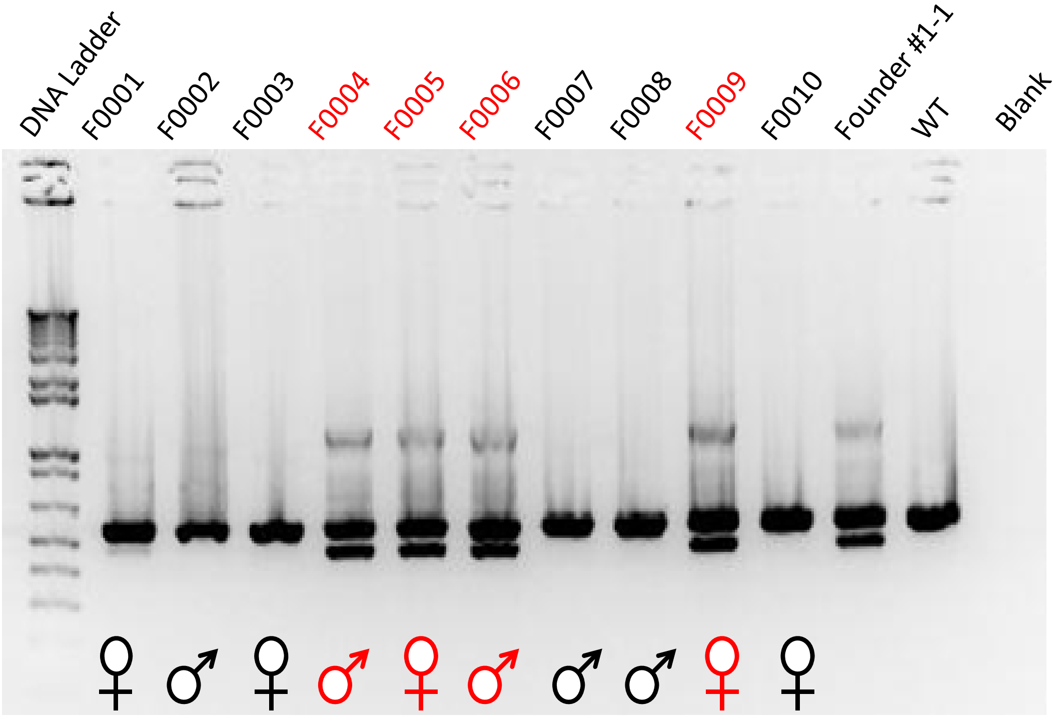

Supplement: Supplementary Information — Supp information [file srep05290-s1.pdf]
